# Adenosine deaminase negative pleural tuberculosis: a case report

**DOI:** 10.1186/s12879-021-06276-4

**Published:** 2021-06-15

**Authors:** Zachary H. Boggs, Scott Heysell, Joshua Eby, Christopher Arnold

**Affiliations:** University of Virginia, Charlottesville, VA 22903 USA

**Keywords:** Tuberculosis, Adenosine deaminase, Pleural effusion, IGRA, Case report

## Abstract

**Background:**

A pleural fluid adenosine deaminase (ADA) has been used globally to assist in the diagnosis of a tuberculous pleural effusion (TPE) with a notable negative predictive value.

**Case presentation:**

We report a case of a patient with a negative pleural fluid ADA who was found to have culture-positive and biopsy-proven *Mycobacterium tuberculosis.*

**Conclusions:**

This case shows the importance of pursuing gold standard diagnostic studies when clinical suspicion remains high despite negative preliminary testing. We further describe gaps in research to improve pleural fluid biomarkers for TPE.

## Background

Tuberculosis (TB) is the world’s leading killer by a single infectious pathogen. Pleural effusions occur in nearly 5% of all *M. tuberculosis* cases [1]. In endemic settings, TB remains the most common cause of a lymphocytic pleural effusion. The gold standard in diagnosis has been through acid fast bacilli (AFB) culture of pleural fluid or through pleural biopsy [2]. Pleural biopsy has been the procedure traditionally used to diagnose a pleural effusion caused by *M. tuberculosis* but carries substantial morbidity. Pleural fluid molecular markers have been developed to forgo invasive testing. Pleural fluid adenosine deaminase (ADA) has a high sensitivity (0.92) and specificity (0.90) for tuberculous pleural effusions (TPE), with a positive likelihood ratio of 9 and a negative likelihood ratio of 0.10 summarized across studies [2–6]. Some have recommended that an ADA level less than 40 IU/L rules out TPE with its exceptional negative predictive value [2].

## Case presentation

**Fig. 1:**
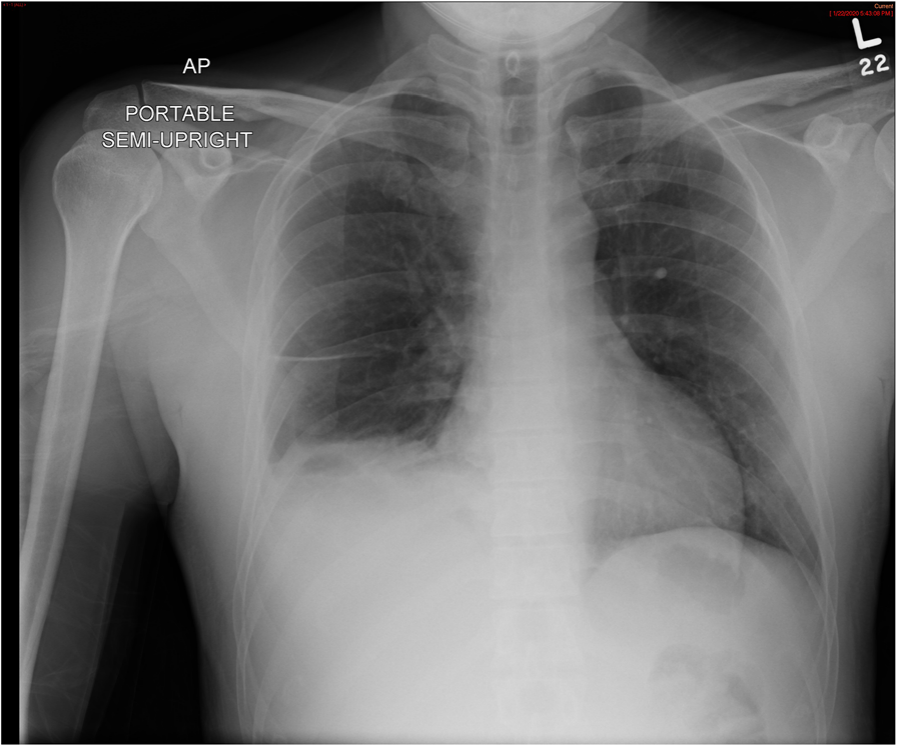
Chest radiograph of the patient showing a right pleural effusion

We present a case of a human immunodeficiency virus-negative 37-year-old man with 4 months of an intermittent, non-productive cough with associated weight loss (body mass index of 19). He had recently spent 6 weeks in the Amazonian jungle and had traveled to Botswana and India within 2 years of presentation. His brother had been previously treated with active TB. His symptoms progressed with the onset of chest discomfort prompting medical evaluation. A chest x-ray showed a right pleural effusion without other pulmonary nodules (Fig. 1). The initial pleural fluid studies showed an exudative process by Light’s criteria and a cell count was not obtained. Bacterial, fungal and AFB cultures were collected, with no elements seen on initial Gram’s stain or AFB smear. He was discharged on azithromycin for presumed parapneumonic effusion. He returned to the hospital 6 days later with increased chest pain and fevers. Repeat thoracentesis demonstrated pleural fluid with 1900 white blood cells/μL (25% polymorphonuclear leukocytes, 54% atypical lymphocytes and 21% lymphocytes). Pleural lactate dehydrogenase was 1121 units/L, glucose 86 mg/dL, pH 7.5 and total protein 5.0 g/dL. Repeat pleural fluid bacterial, fungal and AFB cultures (two slants on Lowenstein-Jensen media, one in liquid media with Mycobacterium Growth Indicator) were obtained and again no organisms were seen on Gram’s stain or AFB smear. Nucleic acid amplification testing (NAAT) for *M. tuberculosis* on the pleural sample was negative (Mayo MTBRP). Sputum AFB cultures revealed no growth. Sputum NAAT (Cepheid GeneXpert MTB/RIF) for TB was negative. A pleural fluid ADA (ARUP 3002978) returned at 6.5 IU/L by quantitative spectrophotometry. Given the very low ADA value, negative NAAT and that cultures of the pleural fluid were preliminarily without growth, alternative diagnoses were considered, and anti-TB therapy was withheld. The otherwise more exhaustive diagnostics included a multiplexed respiratory virus polymerase chain reaction panel, urine antigen studies for *Histoplasmosis* and *Blastomyces* species, and autoimmune serologies; all of which were unremarkable. While computed tomography imaging did not show enlarged lymph nodes to suggest lymphoma and pleural cytology did not demonstrate malignant cells, there were a few small calcified supraclavicular lymph nodes and thus given the very low ADA value and lack of alternate diagnosis, a thoracoscopic pleural biopsy was performed. On hospital day 21, his initial pleural fluid cultures grew *M. tuberculosis*, and later his pleural biopsy also grew *M. tuberculosis.* While awaiting the diagnostic work-up and without anti-TB therapy, he received numerous administrations of antipyretics and continued to lose weight. Once the diagnosis of TB was ultimately made, he was initiated on typical four-drug treatment for drug-susceptible TB. However, he subsequently developed hepatoxicity and required treatment interruption before continuing a triple-drug, pyrazinamide-sparing regimen necessitating an extended total duration. His total hospital course was 30 days, and now has completed treatment. He is clinically improved to near baseline health with a resolved pleural effusion by repeat imaging.

## Discussion

A pleural fluid ADA, given its negative predictive value, is used worldwide to assist in the diagnosis of TPE. AFB cultures often take several weeks to return positive and thoracoscopic biopsy is invasive, holds inherent procedural risks, and is frequently unavailable in TB-endemic settings. Multiple studies have shown that a pleural ADA can aid in TPE diagnosis, while a low level (less than 40 IU/L) is effective in ruling out TPE, even in low prevalence areas [5–9]. However, most studies were conducted in high incidence settings and had a high risk for bias [6]. False negative tests have been implicated in early infection as ADA is not active until there is lymphocytic proliferation, however the patient described in this case had symptoms of TB including prominent weight loss and pleurisy for months prior to the pleural fluid ADA testing [10]. Furthermore, advanced age and tobacco use have been shown to cause a lower pleural ADA in the setting of TPE [11, 12]. The patient was young and a non-smoker and should not have had other reasons to have a blunted ADA response. Common variable immune deficiency could lead to a reduced ADA level, however this patient’s IgG levels were tested and normal. Thus, this case demonstrates that ADA should not remain the sole diagnostic test to rule-out TPE when there remains a high suspicion, and this may be particularly problematic in TB endemic settings if the use of ADA as a negative predictor was used to withhold empiric TB treatment.

Despite the incorporation of ADA into diagnostic algorithms for TPE, the diagnosis remains challenging (Table 1). Pleural interferon-gamma release assays (IGRA), which test fluid interferon-gamma production when stimulated with *M. tuberculosis* specific antigens, have been studied in recent years as potential tests to rapidly rule in or rule out a TPE, however meta-analyses have shown variable diagnostic accuracy [14–16]. Nucleic acid amplification tests have revolutionized sputum-based diagnostics for pulmonary TB but are less sensitive for TPE where the bacillary burden is often low [17]. A recent metanalysis has shown that GeneXpert testing has low sensitivity but high specificity in the evaluation of a TPE, thus may have some utility when positive [18]. Xpert MTB/Rif Ultra has been shown to have a high sensitivity in all specimens including cerebrospinal fluid in the diagnosis of TB meningitis, thus has potential for higher yield in pleural fluid compared to conventional Xpert MTB/Rif [20]. Other novel pleural markers are being studied, including interleukin-27, which has been shown in one meta-analysis to be more specific and sensitive than ADA or IGRA testing [19]. Some research groups have considered diagnostic models of multiple pleural biomarkers in combination to accurately diagnose TPE. Ultimately, more research is needed prior to using these tests to make treatment decisions in the absence of AFB culture or pleural biopsy, and pragmatic trials would advance the field beyond the current excess of diagnostic accuracy studies.

**Table 1:** This table summarizes the meta-analyses for multiple biomarkers and rapid tests on pleural fluid to assist in the diagnosis of TPE. ADA = Adenosine Deaminase; IFNγ = Interferon Gamma; NAAT = Nucleic Acid Amplification Test; Xpert MTB-Rif = Xpert *Mycobacterium tuberculosis* Complex and Resistance to Rifampin Assay; IL-27 = Interleukin-27; LR = Likelihood Ratio

Biomarker	Study	Sensitivity	Specificity	+LR	-LR	Summary
ADA	Liang et al. 2008 [4]	0.92	0.90	9.03	0.10	ADA useful in diagnosis of TPE
	Gui et al. 2014 [13]	0.86	0.88	6.32	0.15	ADA is relatively accurate
	Aggarwal et al. 2019 [6]	0.92	0.90	8.92	0.09	Good accuracy regardless of prevalence or setting
IFNγ	Jiang et al. 2007 [14]	0.89	0.97	23.45	0.11	Relatively accurate
	Zhou et al. 2011 [15]	0.75	0.82	3.49	0.24	Not a useful test
	Aggarwal et al. 2015 [16]	0.72	0.79	3.65	0.31	Not a useful test
NAAT	Pai et al. 2004 [17]	0.62	0.98	25.4	0.40	Unclear utility in TPE
Xpert MTB-Rif	Sehgal et al. 2016 [18]	0.51	0.99	36.7	0.49	Possibly useful when positive
IL-27	Liu et al. 2018 [19]	0.93	0.97	25.88	0.07	Possible novel biomarker

## Conclusions

Diagnosing and confirming TPE can be difficult as pleural culture can take weeks and biopsy is invasive or may be unavailable in TB endemic settings. While pleural fluid ADA has been shown previously to have an excellent negative predictive value, we present a case of ADA-negative TPE without risk factors for a falsely low ADA value. In a patient with a high likelihood of TB, a pleural fluid ADA should not be used exclusively to rule-out TPE. Further research should focus on the predictive accuracy of multiple pleural fluid biomarkers and pragmatic trials of TB treatment initiation.

## Data Availability

All data generated or analyzed during this study are included in this published article.

## Abbreviations

TPE: Tuberculous pleural effusion
ADA: Adenosine deaminase
TB: Tuberculosis
AFB: Acid fast bacilli
IGRA: Interferon-gamma release assays
IL-27: Interleukin-27
NAAT: Nucleic acid amplification test
IFNγ: Interferon gamma
LR: Likelihood ratio
ARUP: Associated regional and university pathologists Lab
Xpert MTB-Rif: Xpert *mycobacterium tuberculosis* complex and resistance to rifampin assay
